# Synergistic Effects of Unmodified Tea Leaves and Tea Biochar Application on Remediation of Cr-Contaminated Soil

**DOI:** 10.3390/toxics12120888

**Published:** 2024-12-06

**Authors:** Weili Qi, Yun Yang, Yan Xu, Xiaowen Teng, Jiawei Ma, Weijie Xu, Zhengqian Ye, Xianzhi Fang, Dan Liu

**Affiliations:** 1 State Key Laboratory of Subtropical Silviculture, Zhejiang A & F University, Hangzhou 311300, China; qwl@stu.zafu.edu.cn (W.Q.); 18868195939@163.com (Y.Y.);; 2 Key Laboratory of Soil Remediation and Quality Improvement in Zhejiang Province, Hangzhou 311300, China; 3 College of Tea Science and Tea Culture, Zhejiang A & F University, Hangzhou 311300, China

**Keywords:** hexavalent chromium, tea biochar, soil remediation, chromium fractionation, bioavailability reduction

## Abstract

Hexavalent chromium (Cr(VI)) contamination in soil presents significant risks due to its high toxicity to both the environment and human health. Renewable, low-cost natural materials offer promising solutions for Cr(VI) reduction and soil remediation. However, the effects of unmodified tea leaves and tea-derived biochar on chromium-contaminated soils remain inadequately understood. In this study, tea tree pruning waste was converted into biochar at various temperatures, and the impacts of both unmodified tea leaves and tea biochar on soil Cr(VI) content, chromium fractionation, and soil biochemical properties were assessed using a soil incubation experiment. The results showed that the combined treatment of tea and tea biochar produced at 500 °C reduced Cr(VI) content by up to 49.30% compared to the control. Chromium fractionation analysis revealed a significant increase in the residual chromium fraction, accounting for 32.97% of total chromium, substantially reducing its bioavailability and mobility. Soil properties were markedly improved, with notable increases in pH (14.89%), cation exchange capacity (CEC; up to 100.24%), and organic matter content (up to 167.12%) under the combined treatments. Correlation analysis confirmed that Cr(VI) content reductions were positively correlated with increases in pH, nutrient retention, and enzyme activities, highlighting their role in chromium stabilization. This study underscores the synergistic potential of unmodified tea leaves and tea biochar as an innovative, eco-friendly strategy for Cr(VI) remediation, enhancing both soil quality and heavy metal stabilization.

## 1. Introduction

The predominant valence states of Cr in soil are hexavalent chromium (Cr(VI)) and trivalent chromium (Cr(III)). Cr(VI) is more toxic than Cr(III) due to its high solubility and mobility. Cr(VI) is a by-product of numerous industrial processes, including leather production, chromium plating, stainless steel welding, pigment production, and even the manufacture of nuclear weapons [1]. The area of land contaminated by Cr in China accounts for more than 5% of the total area of land contaminated by potentially toxic elements, of which Cr pollution exceeds the risk value of the national soil sampling point by 1.10% [2]. Chromium is absorbed by crops and accumulates in plant tissues, which not only affects plant growth and development but also potentially enters the food chain, posing a serious threat to human health [3]. Therefore, the remediation of Cr-contaminated soil is of great significance for the sustainable development of human society.

Reducing Cr(VI) to Cr(III) is a crucial strategy to remediate Cr-contaminated soil to meet relevant regulatory standards and protect human health. Cr(VI) is highly soluble, mobile, and bioavailable in soils, making it more toxic and carcinogenic compared to Cr(III) [4]. The oxidation–reduction conditions in the soil significantly influence the speciation and mobility of chromium. Under oxidizing conditions, Cr exists mainly in the hexavalent state, while reducing conditions favor the formation of trivalent chromium [5]. Currently, the remediation methods for Cr(VI)-contaminated soil mainly include biological reduction and chemical reduction. Bioreduction methods rely on microorganisms to reduce Cr(VI) to the less toxic Cr(III) during metabolic processes, showing the advantages of being environmentally friendly and cost-effective [6]. However, the slow degradation rate of bioreduction methods limits their application in certain scenarios where rapid attenuation of Cr(VI) toxicity is needed. In contrast, chemical reductions are the most widely used because of their high efficiency, adaptability, and low cost. Chemical reductants can alter the redox conditions of the soil microenvironment, promoting the reduction in Cr(VI) to Cr(III) and subsequently affecting its bioavailability and bioaccessibility [7]. Chemical reduction technologies can convert Cr(VI) to Cr(III) and decrease the content, bioavailability, and bioaccessibility of Cr(VI) in soil by adding exogenous reductive agents into Cr-contaminated soil [8]. A variety of materials have been designed and evaluated as excellent chemical reductants, which can be classified into three categories: iron-based reductants, sulfur-based compounds, and organic amendments [2,9]. In recent years, natural organic substances have been increasingly selected as soil remediation alternatives because they are more sustainable, environmentally friendly, inexpensive, and can be reused as agricultural organic residues [10,11]. These organic amendments not only provide electrons for the reduction in Cr(VI) but also improve soil properties such as organic matter content and cation exchange capacity (CEC), which can enhance the adsorption and immobilization of chromium, thereby reducing its bioavailability and bioaccessibility to plants and microorganisms [12]. In addition, considering that reduced Cr(III) may be reoxidized to Cr(VI) under oxidizing conditions, increasing attention has been paid to solving the problem of Cr(III) fixation during soil Cr remediation [13].

As a typical natural organic material, biochar resources are extremely abundant and can be obtained from a variety of agricultural wastes. Biochar application can modify the soil’s redox conditions due to its redox-active functional groups, influencing chromium speciation [14]. Biochar contains multiple active functional groups, such as carboxyl, hydroxyl, and phenolic groups, which could combine with toxic metals and thus reduce their mobility in soil [15,16]. For instance, the biochar derived from sugar beet tailings exhibits a prominent capacity to adsorb Cr (VI) from contaminated water [17]. Moreover, biochar is also involved in converting heavy metal ions into less harmful forms, such as reducing Cr(VI) to Cr(III) [18] and oxidizing As(III) to As(V) [19]. The porous structure of biochar increases the surface area for adsorption, while its functional groups can bind with chromium ions, decreasing their bioavailability and bioaccessibility. Additionally, biochar can enhance microbial activity by providing a habitat for microorganisms that can further contribute to reducing Cr(VI) [20]. Recent studies have highlighted the potential of tea-derived biochar as an economical and environmentally friendly adsorbent for heavy metal remediation. For instance, a composite of tea waste biochar and zero-valent iron has shown high adsorption and reduction capacities for Cr(VI) in contaminated water utilizing the porous structure and active surface groups of the composite [21]. Fan et al. [22] found that the functional groups in tea waste biochar (300 °C) could participate in Cd removal, and the aromatic structures in tea waste biochar (700 °C) may be involved in Cd adsorption. Mandal et al. [23] reported that unmodified tea leaves biochar loaded nano zero-valent iron significantly immobilized Pb and reduced Pb mobility in soil. These findings suggest that tea-derived biochar not only affects the redox conditions but also reduces the bioavailability and mobility of heavy metals through adsorption and complexation mechanisms. These findings imply that biochar prepared from tea waste may play an important role in the process of Cr immobilization and reduction in soil; however, this speculation needs to be further confirmed by experiments.

In addition, some organic materials rich in various metabolic substances, such as polyphenols, citric acid, tartaric acid, gallic acid, and tannic acid, have been shown to effectively reduce Cr(VI) in contaminated soil and water [24,25]. Jiang et al. [26] demonstrated the efficacy of natural polyphenols in converting Cr(VI) to Cr(III) in soil. Gallic acid has been found to reduce Cr(VI) in contaminated water through the formation of a dichromate ester and electron transfer from phenolic groups [27]. Similarly, Zhang et al. [28] reported that tartaric acid effectively reduces Cr(VI) in soil. Anthocyanins, as plant polyphenols, are known to chelate chromium, reducing its toxicity to plants [29]. Generally, plant polyphenols play a key role in Cr remediation through adsorption, reduction, and complexation mechanisms [30]. Tea pruning wastes are valuable resources, rich in functional groups like carboxylates and phenolic hydroxyls, which can form stable complexes with metals like Ni(II), As(III), and Pb(II) [31]. Recent studies have shown that tea polyphenols act as effective and eco-friendly adsorbents for heavy metals [32], with gallic and tartaric acids particularly capable of converting Cr(VI) to Cr(III) [33]. These findings highlight the potential of tea-derived biochar and polyphenols as sustainable agents for reducing chromium toxicity in contaminated soils [26].

Building on the extensively documented roles of biochar and plant polyphenols in the adsorption, fixation, and reduction in heavy metals, this study introduces a novel remediation strategy by combining tea branch biochar and unmodified tea leaves. This approach leverages the unique chemical and structural properties of both materials to address chromium contamination in soils. Unlike conventional methods, the combined application offers a dual mechanism: biochar’s high surface area and functional groups enhance adsorption and stabilization, while unmodified tea leaves contribute bioactive compounds that promote chromium reduction and microbial activity, thereby improving the overall remediation efficiency and long-term stability. This study aimed to (1) examine the effects of the application of unmodified tea leaves and tea biochar alone as well as their combinations on Cr(VI) reduction in soil by a soil incubation experiment; (2) investigate the response characteristics of soil chemical and biological properties to unmodified tea leaves and tea biochar application.

## 2. Materials and Methods

### 2.1. Preparation of Cr-Contaminated Soil

Soil samples were collected from Zhejiang A&F University in Lin’an District, Hangzhou City, Zhejiang Province, China, with a geographic coordinate at 30°15′57″ N, 119°42′50″ E. Soil samples were then air-dried and sieved through a 2 mm sieve for subsequent experiments. The chemical properties and Cr content of the soil samples were analyzed and shown in Table S1. Cr-contaminated soil with a content of approximately 250 mg kg^−1^ Cr was prepared by adding potassium dichromate solution to the soil. Water was applied weekly to maintain the soil moisture at 60% of the field capacity. After aging for twelve months, the available chromium content in the soil was nearly zero, and the total hexavalent chromium content was 72.90 mg kg^−1^. The soil was then air-dried, sieved through a 10-mesh screen, and thoroughly mixed to ensure uniformity before proceeding with subsequent experiments.

### 2.2. Preparation of Material for Chromium Immobilization

The tea tree pruning waste was divided into two components: unmodified tea leaves and tea branches. The unmodified tea leaves were repeatedly rinsed with deionized water, dried at 75 °C to a constant weight, and ground into powder for reserve. The tea branches were similarly treated and sieved through a 10-mesh sieve. The powdered tea branches were placed in a crucible wrapped with aluminum foil to create a sealed environment and were pyrolyzed in a muffle furnace. The pyrolysis process was conducted at three target temperatures: 300 °C, 500 °C, and 700 °C. The heating rate was set to 10 °C min^−1^, and the material was held at the target temperature for 2 h to ensure full pyrolysis. After cooling the furnace to room temperature, the resulting biochar samples were collected and labeled as TB300, TB500, and TB700, corresponding to the pyrolysis temperatures.

### 2.3. Remediation of Cr-Contaminated Soil

To examine the effects of unmodified tea leaves and tea biochar on Cr immobilization efficiency, four treatments were designed: 1% unmodified tea leaves (G), 1% TB300 (B1), 1% TB500 (B2), and 1% TB700 (B3). To examine the combined effects of unmodified tea leaves and tea biochar on Cr immobilization efficiency, three treatments were designed: 1% unmodified tea leaves + 1% TB300 (GB1), 1% unmodified tea leaves + 1% TB500 (GB2), and 1% unmodified tea leaves + 1% TB700 (GB3). CK was set as a control group without unmodified tea leaves and tea biochar. Three replicates were set for each treatment. To a plastic pot, 1 kg of air-dried soil evenly mixed with unmodified tea leaves, tea biochar, or a combination of both according to the mass ratio was added. The soil water contents were adjusted to 60% of the field capacity. After 30 days of cultivation, Cr-contaminated soil samples were collected, and then the soil’s chemical and biological properties and Cr fractions were analyzed.

### 2.4. Analysis of Soil Chemical Properties

Soil pH was determined in a soil solution with a 1:2.5 soil-to-water ratio using a pH meter (FiveEasy Plus; Mettler Toledo, Columbus, OH, USA). Cation exchange capacity (CEC) was measured using the ammonium acetate exchange method and determined using a UV–visible spectrophotometer (T9; Persee, Beijing, China). The organic matter content was determined using the potassium dichromate–sulfuric acid method: after heating the mixture, 0.5 mol L^−1^ ammonium ferrous sulfate solution was used for titration, and the organic matter content was calculated. Ammonium acetate (0.1 mol L^−1^) was used to extract soil-available potassium, and flame photometry was used to determine available potassium content. The available potassium content in the soils were determined using an atomic absorption spectrophotometer (TAS-990AFG; Persee, Beijing, China). Soil-available phosphorus was extracted using 0.03 mol L^−1^ hydrochloric acid-ammonium fluoride solution and analyzed following the Bray method. The available phosphorus concentrations were analyzed using the molybdenum blue method and determined using a UV–visible spectrophotometer (T9; Persee, Beijing, China) [34]. Soil alkali-hydrolyzable nitrogen was extracted using 2.0 mol L^−1^ NaOH solution at a soil-to-solution ratio of 1:2. Then, 2% boric acid was used to absorb the released ammonia, and the residual solution was titrated using 0.01 mol L^−1^ sulfuric acid.

### 2.5. Analysis of Soil Enzyme Activity

The activities of urease, catalase, sucrase, and alkaline phosphatase were determined as described by Feng et al. [35]. Soil urease activity was determined using the colorimetric method with sodium phenol-sodium hypochlorite. Soil catalase activity was determined by potassium permanganate titration. Determination of alkaline phosphatase activity in soil by colorimetric method with disodium benzene phosphate. Determination of soil sucrase activity by colorimetric method with 3,5-dinitrosalicylic acid. All enzyme activities were analyzed using a UV–visible spectrophotometer (T9; Persee, Beijing, China).

### 2.6. Determination of Soil Cr Content and Cr Fractions

The total Cr(VI) content in the soil was quantified using the alkaline digestion method. The extracted Cr(VI) reacted with diphenylcarbazide to form a colored complex, and its absorbance was measured spectrophotometrically at approximately 540 nm to determine the Cr(VI) content in the soil samples [36]. The sequential extraction procedure was used to measure the operational speciation of Cr in soil, namely the weak acid extractable fraction (F1, the most active and potentially toxic fraction), the reducible fraction (F2, bound with Fe/Mn oxides), the oxidizable fraction (F3, bound with organics and sulfides) and the residual fraction (F4, the most stable fraction) [37]. The Cr contents were determined with atomic absorption spectroscopy GFAAS (SHIMADZU AA7000, Kyoto, Japan).

### 2.7. Data Analysis

The data were analyzed using one-way analysis of variance (ANOVA) with the LSD test by the Microsoft Excel 2016 and SPSS21 software. The resulting data were then plotted using Origin 2021 software. Three replicates were set for each treatment. All data are presented as mean ± standard deviation (SD). Differences were considered statistically significant at *p* < 0.05.

## 3. Results

### 3.1. Effects of Unmodified Tea Leaves and Tea Biochar on Soil Chemical Properties

**Figure 1:**
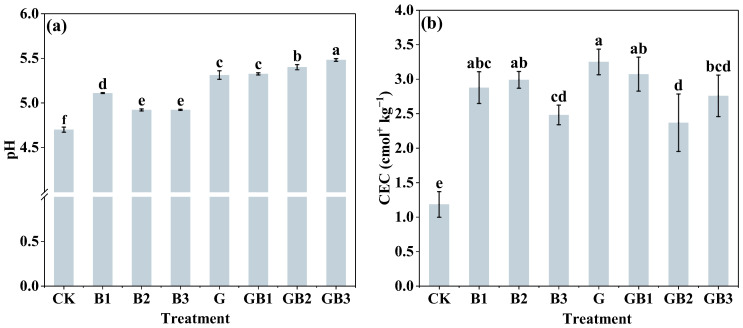
Effect of unmodified tea leaves and tea biochar addition on soil pH and CEC. (**a**) pH; (**b**) CEC. B1: 1% tea biochar prepared at 300 °C; B2: 1% tea biochar prepared at 500 °C; B3: 1% tea biochar prepared at 700 °C; GB1: 1% unmodified tea leaves + 1% BC300; GB2: 1% unmodified tea leaves + 1% BC500; GB3: 1% unmodified tea leaves + 1% BC700; CK: without unmodified tea leaves and tea biochar. Values are the mean of three independent replicates ± standard deviation. Different letters above the bars indicate significant differences among treatments at *p* < 0.05 according to the one-way analysis of variance (ANOVA) with the LSD test.

**Figure 2:**
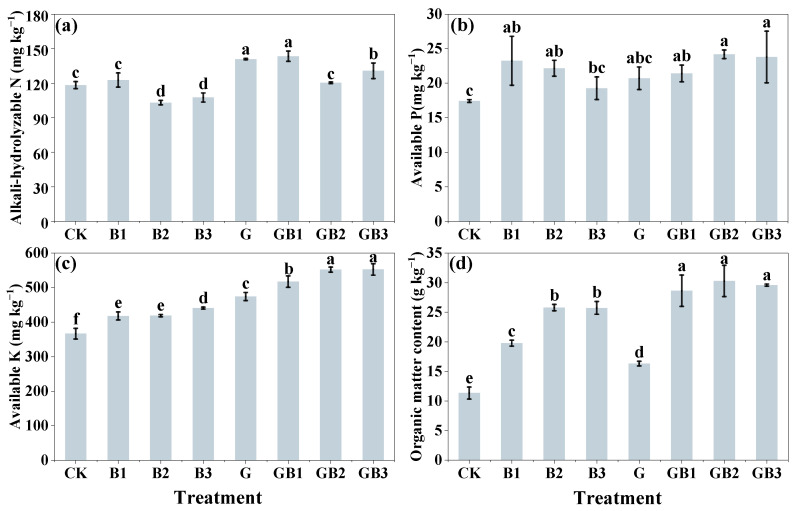
Effect of unmodified tea leaves and tea biochar addition on the contents of nitrogen, phosphorus, potassium, and organic matter of soil. (**a**) AN (mg kg^−1^); (**b**) AP (mg kg^−1^); (**c**) AK (mg kg^−1^); (**d**) SOM (g kg^−1^). B1: 1% tea biochar prepared at 300 °C; B2: 1% tea biochar prepared at 500 °C; B3: 1% tea biochar prepared at 700 °C; GB1: 1% unmodified tea leaves + 1% BC300; GB2: 1% unmodified tea leaves + 1% BC500; GB3: 1% unmodified tea leaves + 1% BC700; CK: without unmodified tea leaves and tea biochar. Values are the mean of three independent replicates ± standard deviation. Different letters above the bars indicate significant differences among treatments at *p* < 0.05 according to the one-way analysis of variance (ANOVA) with the LSD test.

Considering that the alteration of soil properties could affect the availability of heavy metals, we first compared the effects of unmodified tea leaves and tea biochar application on soil chemical properties. As shown in Figure 1a, the application of unmodified tea leaves alone significantly increased soil pH by 13.02% (*p* < 0.05), while all three biochar (B1–B3) alone treatments resulted in a slight elevation in soil pH, ranging from 4.70 to 8.72%, compared with the control. Among the three biochar treatments, B1 (300 °C) exhibited the greatest increase in soil pH, followed by B2 (500 °C) and B3 (700 °C). This indicated that lower pyrolysis temperatures (300 °C) might be more effective in retaining functional groups contributing to pH regulation. In particular, soil pH was increased by 0.25–3.18% and 4.22–11.35% by the combined addition of unmodified tea leaves and tea biochar compared with the single addition of unmodified tea leaves and tea biochar, respectively. In addition, unmodified tea leaves and tea biochar application alone led to a significant increase of 174.72%, 143.20%, 152.75%, and 129.88% in CEC content of the soil, respectively, compared with the control (*p* < 0.05). B1 (500 °C) demonstrated the highest CEC enhancement among the three biochar treatments, likely due to its retention of acidic functional groups, which facilitate cation exchange. Soil CEC content was significantly enhanced by 100.24–159.70% under the combined treatment of unmodified tea leaves and biochar (GB1-GB3) compared to that in the control (*p* < 0.05; Figure 1b). We next examined whether unmodified tea leaves and tea biochar could affect the contents of nutrients and organic matter in the soil. The addition of tea biochar (B1–B3) significantly enhanced the contents of available phosphorus, available potassium, and organic matter by 10.63–33.49%, 13.96–20.17%, and 74.43–127.44% of soil, respectively, compared to the control (*p* < 0.05; Figure 2a–d). Among the three biochars, B3 (700 °C) demonstrated the greatest improvement in organic matter content (127.44%) and available potassium (20.17%). However, for available phosphorus, B1 (300 °C) showed the highest enhancement (33.49%) among biochar treatments. Similarly, the contents of alkali-hydrolyzed nitrogen, available potassium, and organic matter of soil under the treatment of unmodified tea leaves (G) addition alone were 19.09%, 29.29%, and 43.82% higher than those in the control, respectively. The alkali-hydrolyzed nitrogen, available phosphorus, available potassium, and organic matter of soil were significantly enhanced by 10.54%, 21.24% (GB1 and GB3), 22.89–38.81% (GB1–GB3), by 41.12–50.71% (GB1–GB3), and by 152.55–167.12% (GB1–GB3), respectively, compared with the control (*p* < 0.05; Figure 2a–d). In comparison with unmodified tea leaves application alone, the combined application of unmodified tea leaves and tea biochar significantly increased soil available potassium and organic matter content by 9.15–16.57% and 75.61–85.73% (*p* < 0.05), respectively. The results indicate that unmodified tea leaves and tea biochar application increased the nutrient contents of Cr-contaminated soil.

### 3.2. Unmodified Tea Leaves and Tea Biochar Application Altered Soil Enzyme Activities

**Figure 3:**
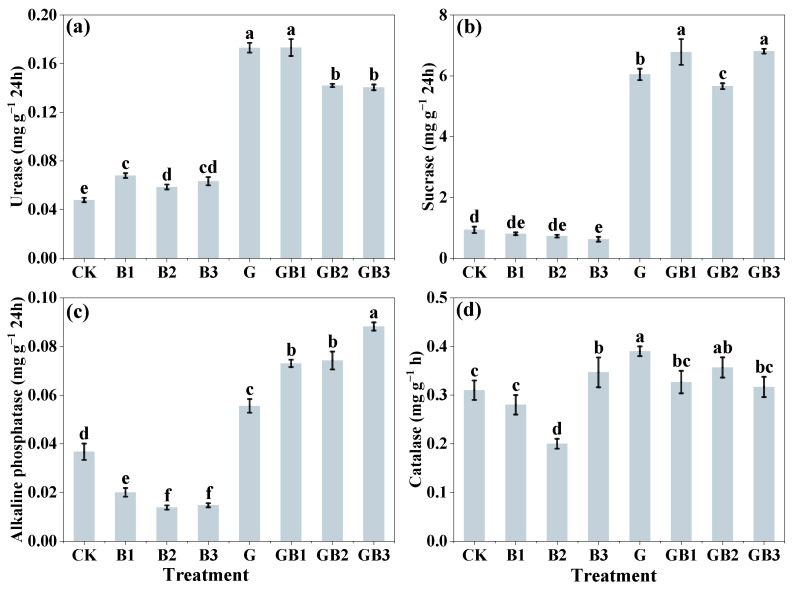
Effect of unmodified tea leaves and tea biochar addition on soil enzyme activity. (**a**) Urease (mg g^−1^ 24 h); (**b**) sucrase (mg g^−1^ 24 h); (**c**) acid phosphatase (mg g^−1^ 24 h); (**d**) catalase (mg g^−1^ h). B1: 1% tea biochar prepared at 300 °C; B2: 1% tea biochar prepared at 500 °C; B3: 1% tea biochar prepared at 700 °C; GB1: 1% unmodified tea leaves + 1% BC300; GB2: 1% unmodified tea leaves + 1% BC500; GB3: 1% unmodified tea leaves + 1% BC700; CK: without tea and tea biochar. Values are the mean of three independent replicates ± standard deviation. Different letters above the bars indicate significant differences among treatments at *p* < 0.05 according to the one-way analysis of variance (ANOVA) with the LSD test.

Soil enzymes play a crucial role in element speciation transformation within the soil. We further investigated the effects of unmodified tea leaves and tea biochar addition on soil enzyme activities. The results showed that, compared to the control, the addition of tea biochar alone significantly increased urease activity by 22.46–42.05% in soil (*p* < 0.05; Figure 3a). However, the tea biochar application alone led to a notable reduction in the activities of sucrase, alkaline phosphatase, and catalase by 33.28% (B3, 700 °C), 45.48% to 62.47% (B1–B3), and 35.48% (B2, 500 °C), respectively (*p* < 0.05; Figure 3b–d). In contrast, the unmodified tea leaves application alone resulted in a significant increase in soil urease, sucrase, alkaline phosphatase, and catalase activities by 261.39%, 547.35%, 51.39%, and 25.81% (*p* < 0.05), respectively, compared to the control. Additionally, we found that the combined application of unmodified tea leaves and tea biochar further enhanced the activities of urease, sucrase, and alkaline phosphatase by 193.43–261.70%, 505.79–628.92%, and 98.99–140.36% compared with the control (*p* < 0.05; Figure 3a–d). These results suggest that the application of unmodified tea leaves alone or in combination with tea biochar increased enzyme activities of Cr-contaminated soil.

### 3.3. Effects of Unmodified Tea Leaves and Tea Biochar on Cr(VI) Content in Soil

**Figure 4:**
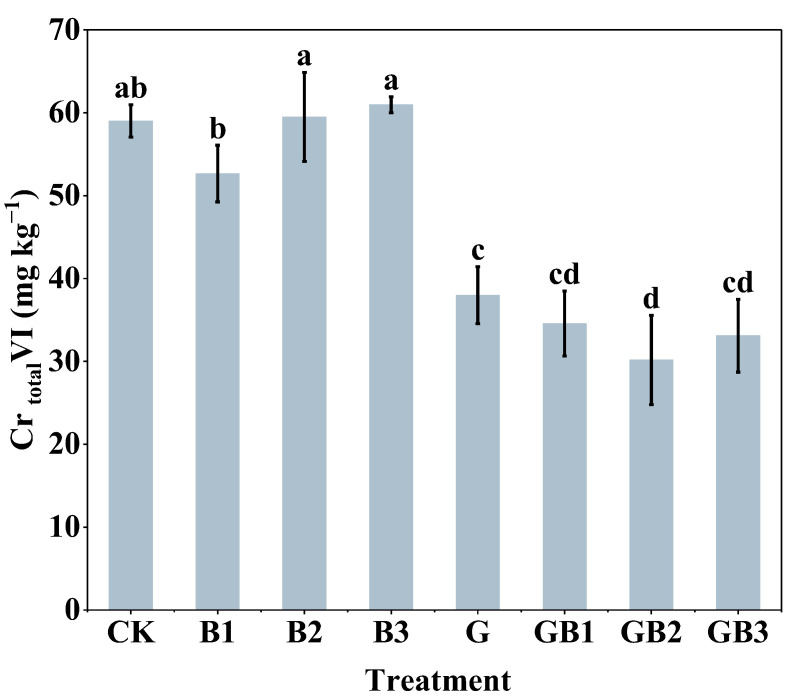
Effects of unmodified tea leaves and tea biochar addition on the total amount of hexavalent chromium in soil. Total Cr(VI) content. B1: 1% tea biochar prepared at 300 °C; B2: 1% tea biochar prepared at 500 °C; B3: 1% tea biochar prepared at 700 °C; GB1: 1% unmodified tea leaves + 1% BC300; GB2: 1% unmodified tea leaves + 1% BC500; GB3: 1% unmodified tea leaves + 1% BC700; CK: without unmodified tea leaves and tea biochar. Values are the mean of three independent replicates ± standard deviation. Different letters above the bars indicate significant differences among treatments at *p* < 0.05 according to the one-way analysis of variance (ANOVA) with the LSD test.

This study further investigated the effectiveness of the application of unmodified tea leaves and tea biochar on the remediation of Cr-contaminated soils. In comparison with the control group, the unmodified tea leaves treatment alone resulted in a significant reduction of 35.62% in Cr(VI) content, while the combined treatments of unmodified tea leaves and tea biochar significantly decreased Cr(VI) content by 41.42% (GB1, 300 °C), 48.88% (GB2, 500 °C), and 43.91% (GB3, 700 °C), respectively (*p* < 0.05; Figure 4). It was observed that the application of biochar prepared at 300 °C reduced the content of Cr(VI) by 10.77% in soil. However, there was no significant impact of tea biochar at different temperatures on soil Cr(VI) content (Figure 4). The combined treatments (GB1–GB3) significantly reduced Cr(VI) levels in soil compared to the biochar-only treatments (B1–B3), with the most effective reduction by GB2 (49.30%) (*p* < 0.05). The results demonstrate that applying unmodified tea leaves alone or in combination with tea biochar efficiently reduced Cr(VI) content in soil.

### 3.4. Effects of Unmodified Tea Leaves and Tea Biochar on Cr Chemical Speciation in Soil

**Figure 5:**
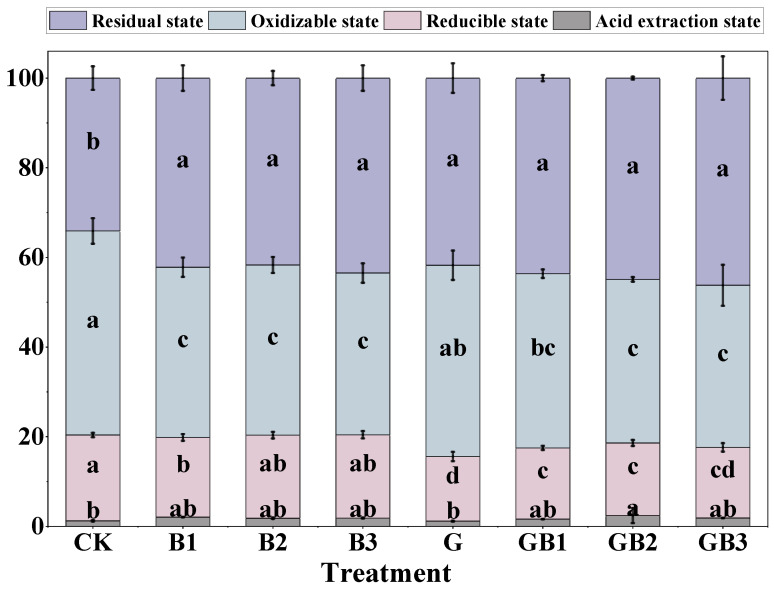
Effects of unmodified tea leaves and tea biochar addition on the total amount of four chromium forms in soil. Purple: residual fraction (F1); blue: oxidizable fraction (F2); pink: reducible fraction (F3); gray: acid-extractable fraction (F4). The different treatments in the figure are the same as in Figure 4. Values are the mean of three independent replicates ± standard deviation. Different letters in the bars with the same color indicate significant differences among treatments at *p* < 0.05 according to the one-way analysis of variance (ANOVA) with the LSD test.

Chromium speciation in soil helps us understand the stability and bioavailability of Cr, thereby assessing its environmental risk and mobility in the soil. We further investigated the effects of the application of unmodified tea leaves and tea biochar on soil Cr speciation. The results showed that residual chromium, the most stable form of chromium, was significantly increased by 22.28–27.53% in the tea biochar treatments (B1–B3) compared to that in the control (*p* < 0.05). The unmodified tea leaves treatment alone also significantly enhanced residual chromium by 22.83% (*p* < 0.05). Similarly, when unmodified tea leaves and tea biochar were applied together (GB1–GB3), the proportion of residual chromium substantially increased with gains of 28.05–35.55% (Figure 5). Oxidizable chromium (a more reactive form) was notably reduced by 16.58–20.77% in the biochar treatments alone in comparison with the control (*p* < 0.05). The unmodified tea leaves addition alone had little effect on oxidizable chromium content (Figure 5). However, the combined treatments (GB1–GB3) resulted in a significant reduction in oxidizable chromium compared to the unmodified tea leaves treatment alone, particularly in the GB2 and GB3 treatments, in which the reductions reached 9.19–15.56% (*p* < 0.05; Figure 5). Compared to the control, the application of unmodified tea leaves alone significantly reduced the reducible chromium content in the soil by 24.40% (*p* < 0.05). Meanwhile, the combined application of unmodified tea leaves and tea biochar notably decreased the proportion of reducible chromium in the soil by approximately 14.56% to 21.47% relative to the control (*p* < 0.05). The biochar at different temperatures (B1–B3) had no obvious effect on the content of reducible chromium (Figure 5). The unmodified tea leaves treatment showed no significant change in acid-extractable chromium compared to CK, whereas the combined application of biochar and unmodified tea leaves led to an increase in acid-extractable chromium, with a notable rise of 96.82% under GB2 treatment (*p* < 0.05). These findings indicate that the combination of unmodified tea leaves and tea biochar is more effective in lowering the bioavailability of chromium, thereby enhancing chromium stability in soil.

### 3.5. Correlation Analysis of Chromium and Soil Properties

**Figure 6:**
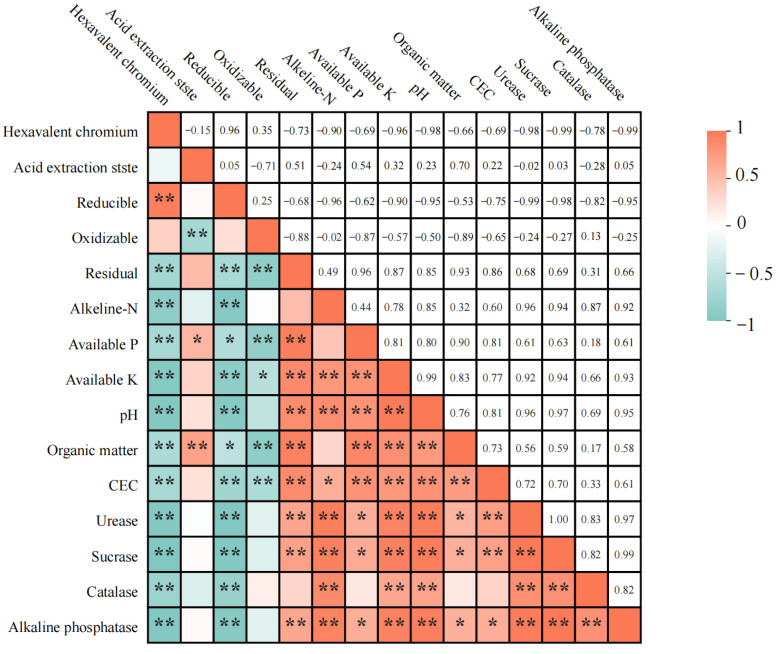
The heatmap shows the Pearson correlation coefficients between chromium speciation (hexavalent, acid-extractable, reducible, oxidizable, and residual) and soil properties (pH, CEC, nutrients, and enzyme activities). Correlation coefficients range from −1 (strong negative correlation, blue) to +1 (strong positive correlation, red). “*” indicates a significant correlation at *p* < 0.05. “**” indicates a highly significant correlation at *p* < 0.01. Non-significant correlations are left unmarked for clarity.

We finally analyzed the correlation between soil environmental factors (e.g., pH, CEC, nutrients, and enzyme activities) and soil Cr speciation under treatment with unmodified tea leaves and tea biochar, which contributed to our understanding of the efficiency of unmodified tea leaves and tea biochar for remediation of Cr-contaminated soils. Figure 6 showed that soil pH exhibited a highly significant negative correlation with Cr(VI) content (correlation coefficient r = −0.98, *p* < 0.01), indicating a decrease in Cr(VI) content induced by a soil pH increase. Soil pH showed a moderate negative correlation with oxidizable chromium (r = −0.57). Conversely, soil pH had a highly significant positive correlation with residual chromium (r = 0.85, *p* < 0.01). Moreover, soil pH exhibited a negative correlation with the content of oxidizable chromium and a highly significant positive correlation with the content of residual chromium, suggesting that higher pH values may facilitate the transformation of chromium into more stable residual forms, thereby increasing the residual chromium content. In addition, CEC showed significant negative correlations with the content of Cr(VI) (r = −0.69, *p* < 0.05), acid-extractable chromium, and oxidizable chromium. It also negatively correlated with reducible chromium (r = −0.75, *p* < 0.01) and oxidizable chromium (r = −0.65, *p* < 0.01), implying that higher CEC reduces the mobility and bioavailability of chromium. A strong positive correlation was observed between CEC and residual chromium (r = 0.86, *p* < 0.01). There was also a significant negative correlation between nutrient indicators (available phosphorus, available potassium, and organic matter) and Cr (VI) content (r = −0.69, −0.96, and −0.66, *p* < 0.05, respectively). Available phosphorus exhibited a significant negative correlation with Cr(VI) content (r = −0.69, *p* < 0.05) and a highly significant positive correlation with residual chromium (r = 0.96, *p* < 0.01). Available potassium was strongly negatively correlated with Cr(VI) content (r = −0.96, *p* < 0.01) and positively correlated with residual chromium (r = 0.87, *p* < 0.01). Organic matter showed a negative correlation with Cr(VI) (r = −0.66, *p* < 0.05) and a highly significant positive correlation with residual chromium (r = 0.93, *p* < 0.01). These nutrient indicators were significantly positively correlated with the content of residual chromium, with correlation coefficients of 0.96, 0.87, and 0.93 (*p* < 0.01), respectively. The findings indicated that higher contents of CEC and nutrients could help reduce chromium bioavailability and promote the conversion of chromium into more stable forms. Similarly, the activities of urease, sucrase, catalase, and phosphatase exhibited highly significant negative correlations with the content of Cr(VI), with correlation coefficients of −0.98, −0.99, −0.78, and −0.99 (*p* < 0.01), respectively, and showed positive correlations with the content of residual chromium, with correlation coefficients of 0.68, 0.69, 0.31, and 0.66, (*p* < 0.05), respectively. Sucrase showed similar patterns, with a negative correlation with Cr(VI) (r = −0.99, *p* < 0.01) and a positive correlation with residual chromium (r = 0.69, *p* < 0.05). Catalase displayed a negative correlation with Cr(VI) (r = −0.78, *p* < 0.01) and a weaker positive correlation with residual chromium (r = 0.31). Alkaline phosphatase showed a highly significant negative correlation with Cr(VI) (r = −0.99, *p* < 0.01) and a significant positive correlation with residual chromium (r = 0.66, *p* < 0.05). This indicated that high enzyme activities may promote the reduction in bioavailable chromium and facilitate its transformation into more stable forms.

## 4. Discussion

The study builds on numerous existing studies demonstrating the potential of organic amendments to mitigate heavy metal contamination in soil. Our findings highlighted the synergistic effects of unmodified tea leaves and tea biochar in reducing chromium bioavailability and promoting soil health [38]. The application of unmodified tea leaves and tea biochar significantly altered soil pH and CEC, which are crucial for controlling heavy metal availability and nutrient uptake. We found that applying unmodified tea leaves or tea biochar alone significantly increased soil pH, whereas a more pronounced elevation of soil pH was observed under the treatment of the combined application of unmodified tea leaves and tea biochar (Figure 1a), indicating a synergistic effect between unmodified tea leaves residues and biochar. The combined application may promote the release of basic cations like calcium, magnesium, and potassium from both materials, contributing to increased soil alkalinity [39]. The increase in soil pH has been reported to facilitate the precipitation of Cr(III) and reduce the solubility of Cr(VI), thereby decreasing Cr mobility and toxicity [40], as confirmed by our results of a strong negative correlation between pH and Cr(VI) content (r = −0.98, *p* < 0.01) (Figure 6). This is consistent with previous studies suggesting that unmodified tea leaves themself may reduce heavy metals through natural organic acids and polyphenols [41], while biochar derived from organic waste has been shown to effectively adsorb and reduce Cr(VI) due to its porous structure and functional groups [42]. Given their biological characteristics, unmodified tea leaves and tea biochar may work synergistically in reducing Cr(VI) and fixing Cr(III). Additionally, the increased soil pH resulting from the amendments facilitated the transformation of chromium into residual species, which was supported by the result that pH was significantly positively correlated with the content of residual chromium (r = 0.85, *p* < 0.05) (Figure 6). Similar findings have been reported in previous studies that alkaline conditions favored chromium immobilization by converting more bioavailable oxidizable chromium into stable residual forms [43]. This is consistent with findings from Omara et al. [44], which suggest that higher-temperature biochars promote the sorption and precipitation processes responsible for stabilizing heavy metals, effectively reducing their bioavailability and mobility. In addition, applying unmodified tea leaves and tea biochar alone or in combination significantly increased CEC in soil (Figure 1b), indicating an increase in the soil fertility potential of unmodified tea leaves and tea biochar. Tea biochar could improve nutrient retention and exchange capacity due to its high surface area and porosity [45]. These properties were enhanced at higher pyrolysis temperatures, which increased the development of micropores and aromatic structures within the biochar [46]. When unmodified tea leaves and tea biochar were used together, it may be beneficial to the formation of stable organic-mineral complexes and, facilitate the retention of cation of soil [47]. The biochar’s properties influenced by its pyrolysis temperature may also affect its interaction with organic matter from the unmodified tea leaves, potentially forming more stable complexes that enhance soil fertility and heavy metal immobilization [48]. A study by Omara et al. [44] also reported that integrating biochar with organic materials could optimize soil structure and nutrient dynamics. We observed that CEC exhibited significant negative correlations with Cr(VI) content, acid-extractable chromium, and oxidizable chromium, which suggested that the elevated soil CEC promoted Cr stabilization, thereby decreasing its mobility and associated environmental risks [39,49].

Soil fertility changes may also affect the availability of soil heavy metals [50]. Our findings showed that the individual application of unmodified tea leaves and tea biochar increased soil nutrients to varying degrees, while their combined application in enhancing soil fertility was more significant (Figure 2a–d). This result can be intuitively explained by the nutritional elements contained in unmodified tea leaves and tea biochar. The combined application of unmodified tea leaves and tea biochar may not only optimize nutrient availability but also promote long-term soil fertility improvement. The high surface area and porous structure of tea biochar could improve nutrient retention by increasing the CEC content and water-holding capacity of the soil, which might help to adsorb Cr(VI) ions [51,52]. These characteristics were dependent on the pyrolysis temperature used during biochar production, as higher temperatures typically result in biochars with greater surface area and porosity [44]. Unmodified tea leaves can both improve soil physical structure and regulate microbial activity by facilitating organic matter decomposition and nutrient cycling, which may indirectly promote the reduction in Cr(VI) to the less toxic Cr(III) [53,54]. Significant positive correlations observed between nutrient levels and residual chromium (e.g., r = 0.96, *p* < 0.05 for phosphorus) support the notion that nutrient enrichment may facilitate the conversion of mobile chromium species into more stable forms [55]. Similarly, Wang et al. [56] found that nutrient-rich conditions in contaminated soils effectively stabilized heavy metals and thus mitigated their ecological risks. These findings underscored the potential of organic amendments to simultaneously enhance soil quality and reduce environmental risks associated with chromium contamination.

Soil enzymes are biocatalysts that produce various biochemical reactions in soil. The combined application of unmodified tea leaves and biochar significantly influenced soil enzyme activities, which play vital roles in microbial metabolism and soil health, particularly in the context of heavy metal remediation [57]. The presence of tea biochar alone notably enhanced urease activity, a key enzyme for nitrogen cycling, whereas the activity of sucrase, alkaline phosphatase, and catalase activities was reduced by tea biochar (Figure 3b–d). The result indicated a selective enhancement in certain microbial functions, particularly those related to nitrogen, over other metabolic pathways [58]. Based on previous studies, it is hypothesized that tea biochar influences microbial communities indirectly by altering soil physical and chemical properties, such as pH and organic matter content. Previous studies suggest that biochar’s porous structure may create favorable microhabitats for microbial colonization, thereby selectively enhancing urease activity due to improved nitrogen availability [59]. Moreover, specific microbial communities, including nitrifying bacteria such as Nitrosomonas and Nitrobacter, have been shown to proliferate in the presence of biochar, which could contribute to increased urease activity [60]. In contrast, the application of unmodified tea leaves alone significantly increased multiple soil enzyme activities, which may be associated with the organic compounds in unmodified tea leaves providing essential nutrients and stimulating a variety of microbial processes [61]. Unmodified tea leaves contain diverse chemical compounds, such as polyphenols, free amino acids, and caffeine, which are known to influence microbial metabolism. Polyphenols in tea leaves can act as substrates or inhibitors for specific soil microorganisms, thereby affecting their growth and enzymatic activities [62]. For instance, catechins and other flavonoids may enhance the growth of beneficial bacteria like Pseudomonas and Bacillus species, which are known to produce enzymes involved in nutrient cycling and heavy metal transformation [63]. Additionally, caffeine can influence microbial community composition by serving as a nitrogen source for certain microbes. These interactions may selectively enrich microbial taxa, including Actinobacteria and fungi, which contribute to nutrient cycling and enzymatic activities [63]. While our study did not directly measure microbial communities, it is hypothesized that the observed increases in enzymatic activities may be linked to these microbial responses to unmodified tea leaf amendments [64]. Future research should focus on characterizing the microbial communities responsible for the observed enzymatic activities to better understand the mechanisms underlying chromium reduction and stabilization. Compared with the individual application, unmodified tea leaves and tea biochar combined application further amplified soil enzyme activities. The surface area of biochar could support microbial colonization, and unmodified tea leaves provide bioavailable organic compounds, which could provide a plausible explanation for the elevated soil enzyme activity [65]. The synergistic effect likely resulted from biochar improving soil structure and microbial habitats, while tea supplied bioavailable organic nutrients to support microbial growth. This suggested a possible interaction where biochar provided a habitat for microorganisms, and the tea leaves supplied nutrients and bioactive compounds that stimulated microbial growth and activity. For example, certain microbial taxa, such as Actinobacteria and Proteobacteria, may be enriched under these conditions, contributing to increased enzymatic activity and heavy metal transformation [66]. Future studies integrating high-throughput sequencing and enzyme profiling could validate these mechanisms and provide a deeper understanding of their interactions. The enhanced activities of urease and phosphatase enzymes were correlated with reduced Cr(VI) levels in soil, suggesting that the increased enzymatic activity may facilitate the transformation of bioavailable Cr into more stable forms. The observed strong negative correlation between enzyme activities and Cr(VI) content (e.g., r = −0.98, *p* < 0.01 for urease) partly supports the hypothesis that enzyme-driven nutrient cycling and organic matter decomposition improves soil sorption capacity, promoting the immobilization of chromium [67]. Moreover, numerous studies have corroborated the indirect relationship between microbial diversity, enzymatic activity, and chromium stabilization. For instance, in tea plantations, an increased microbial diversity enhances soil enzymatic processes, which subsequently facilitates the immobilization of heavy metals [68]. Although microbial diversity was not directly examined in this study, these interactions offer a theoretical foundation for the observed reduction in Cr(VI).This finding is consistent with previous studies that highlight the role of biochar in creating a habitat supportive of microbial processes, which may further benefit from organic amendments to maximize enzyme-driven heavy metal stabilization [69,70]. Therefore, the combined treatment of unmodified tea leaves and tea biochar is a promising approach to enhance soil health and reduce Cr toxicity by promoting enzyme activities that facilitate nutrient cycling and chromium immobilization, thereby mitigating environmental and health risks.

## 5. Conclusions

This study systematically demonstrated the effectiveness of unmodified tea leaves and tea-derived biochar, both individually and in combination, in stabilizing chromium in contaminated soils. The combined application of 1% unmodified tea leaves (GT) and 1% biochar produced at 500 °C (BC500) achieved the highest Cr(VI) removal rate of 49.30%, significantly reducing chromium’s environmental mobility and toxicity. Additionally, treatments with unmodified tea leaves and biochar enhanced soil properties by increasing pH, cation exchange capacity (CEC), organic matter content, and enzyme activities, improving soil fertility. The significant reductions in Cr(VI) levels and improvements in soil properties highlight the potential of using unmodified tea leaves and biochar as sustainable, cost-effective amendments for remediating Cr(VI)-contaminated soils. Future research should focus on characterizing the properties of biochar and unmodified tea to better understand the underlying mechanisms of chromium stabilization. Moreover, validating this method across diverse soil types and environmental conditions would broaden its environmental benefits and practical applications in heavy metal remediation.

## Data Availability

The data presented in this study are available upon request from the corresponding author.

## Supplementary Materials

The following supporting information can be downloaded at www.mdpi.com/article/10.3390/toxics12120888/s1. Table S1: Basic chemical properties of soil.
