# Supplementary material for: Synergistic Effects of Unmodified Tea Leaves and Tea Biochar Application on Remediation of Cr-Contaminated Soil
Source: Toxics. 2024 Dec 6;12(12):888. doi: 10.3390/toxics12120888 (PMC11728837; doi:10.3390/toxics12120888)
Supplement: Supplementary file 1 [file toxics-12-00888-s001.zip › toxics-3346615-supplementary.pdf]

Synergistic Effects of Unmodified Tea Leaves and Tea Biochar Application on Remediation of Cr-Contaminated Soil

Table S1. Basic chemical properties of soil

Characteristic	Unit	Means ± STD
Saturated moisture content	%	31.53 ± 1.60
Field capacity	%	26.20 ± 0.77
pH	—	4.73 ± 0.11
CEC	$cmol^+ kg^{-1}$	1.18 ± 0.10
SOM	$g kg^{-1}$	11.33 ± 0.58
Available N	$mg kg^{-1}$	18.32 ± 1.76
Available P	$mg kg^{-1}$	17.42 ± 0.20
Available K	$mg kg^{-1}$	366.36 ± 9.01
Total Cr	$mg kg^{-1}$	47.72 ± 5.80
Cr(VI)	$mg kg^{-1}$	Not detected

Data represents means ± standard deviation.

Chemical properties and chromium content of soil samples used in the study. The table includes various soil parameters such as saturated moisture content (31.53 ± 1.60%) and field capacity (26.20 ± 0.77%), which reflect the soil's water retention abilities. Soil pH (4.73 ± 0.11) indicates a slightly acidic environment. The CEC is 1.18 ± 0.10 $cmol^+ kg^{-1}$, and soil organic matter content is 11.33 ± 0.58 g kg^{-1}, both of which are important for soil fertility. Available nutrient levels, including available N (18.32 ± 1.76 mg kg^{-1}), available P (17.42 ± 0.20 mg kg^{-1}), and available K (366.36 ± 9.01 mg kg^{-1}), are also listed. The total chromium (Total Cr) content is 47.72 ± 5.80 mg kg^{-1}, while Cr(VI) was not detected, indicating an initial low risk of Cr(VI) toxicity. These properties provide baseline data for subsequent Cr remediation experiments.
